# Robust Feature Selection Approach for Patient Classification using Gene Expression Data

**DOI:** 10.6026/97320630013327

**Published:** 2017-10-31

**Authors:** Md. Shahjaman, Nishith Kumar, Md. Shakil Ahmed, AnjumanAra Begum, S. M. Shahinul Islam, Md. Nurul Haque Mollah

**Affiliations:** 1 Bioinformatics Lab, Department of Statistics, University of Rajshahi-6205, Bangladesh; 2 Department of Statistics, Begum Rokeya University, Rangpur-5400, Bangladesh; 3 Department of Statistics, Bangabandhu Sheikh Mujibur Rahman Science and Technology University, Gopalganj, Bangladesh; 4 Institutitute of Biological Science (IBSc), University of Rajshahi, Rajshahi-6205, Bangladesh

**Keywords:** Feature selection, classification, robust SAM, β-divergence estimators

## Abstract

Patient classification through feature selection (FS) based on gene expression data (GED) has already become popular to the research communities. T-test is the well-known statistical FS method in GED analysis. However, it produces higher false positives and lower accuracies for small sample sizes or in presence of outliers. To get rid from the shortcomings of t-test with small sample sizes, SAM has been applied in GED. But, it is highly sensitive to outliers. Recently, robust SAM using the minimum β-divergence estimators has overcome all the problems of classical t-test & SAM and it has been successfully applied for identification of differentially expressed (DE) genes. But, it was not applied in classification. Therefore, in this paper, we employ robust SAM as a feature selection approach along with classifiers for patient classification. We demonstrate the performance of the robust SAM in a comparison of classical t-test and SAM along with four popular classifiers (LDA, KNN, SVM and naive Bayes) using both simulated and real gene expression datasets. The results obtained from simulation and real data analysis confirm that the performance of the four classifiers improve with robust SAM than the classical t-test and SAM. From a real Colon cancer dataset we identified 21 additional DE genes using robust SAM that were not identified by the classical t-test or SAM. To reveal the biological functions and pathways of these 21 genes, we perform KEGG pathway enrichment analysis and found that these genes are involved in some important pathways related to cancer disease.

## Background

Nowadays the big biological data is one of the hottest topics for the researchers. Gene expression datasets is the high-dimensional big datasets because it contains ten thousands of genes/features with very few patients/samples [1]. This behavior of gene expression data often refers to the curse of dimensionality [2-3]. Thus analyzing of these types of datasets has become complicated and challenging for the researchers. The goal of classification is to allocate/classify the new objects into one of two or more population of the training dataset whose categories are known in advance. Cancer classification based on gene expression dataset is important for subsequent diagnosis and treatment. Without correct classification of different cancer types of the patient, it is very difficult to provide proper treatment and therapies [4]. The conventional classification methods are largely dependent on different morphological parameters to classify cancer. Thus their applications become limited with low prediction accuracies. To get rid from the curse of dimensionality of GED, classification through informative gene identification or feature selection (FS) has already attracted to the research communities [5]. FS can boost the performance of the classifiers by selecting smaller number of features. It also reduces the computational time and provides more reliable estimates to train the classifiers. There are three types of FS methods for GED analysis; (a) wrapper method, (b) embedded method and (b) filter based method [6-7]. Wrapper method searches the features until a certain accuracy of the classifier was achieved. Embedded methods embed feature selection within classifier construction. Filter based method first select few informative features (DE genes) using the labeled samples of training dataset and based on these pre selected features, researchers perform the further classification task. Filter based methods are easily understandable and computationally faster than the wrapper and embedded methods, thus they are better suited to high dimensional datasets [8]. Among the filter-based methods, t-test is one of the popular and widely used methods in gene expression data analysis [9].

However, the major drawback of this classical t-test is that it produces higher false discoveries and lower accuracies with small-sample sizes or outlying gene expressions. Significance Analysis of Microarrays (SAM) has overcome the shortcomings of t-test for small-sample case by controlling false discoveries [10]. However, SAM is very sensitive to outliers and produces misleading results in presence of outlying gene expressions. Consequently, the popular classifiers produce misleading results in presence of outliers when feature selection is performed using classical t-test or SAM. Recently, we have robustified the SAM approach by minimum β-divergence estimators to solve the allaforesaid problems of classical t-test and SAM [11]. Therefore, in this paper, we employ robust SAM as a feature selection method along with classifiers. To investigate the performance of the robust SAM in a comparison with classical t-test and SAM, we pick up four popular classifiers: linear discriminant analysis (LDA) [12], K-nearest neighborhood (KNN) [13], support vector machine (SVM) [14] and naive Bayes classifier [15]. From a real Colon cancer dataset we identified additional 21 DE genes using robust SAM approach that were not identified by the classical ttest or SAM approach. Using the functional annotation and KEGG pathway enrichment analysis we revealed that 15 genes out of 21 genes, are involved in some important pathways related to cancer disease.

## Methodology

### Performance Evaluation

In order to evaluate the performance of different classifiers for binary classification test such as normal or cancer, we used different statistical measures. For binary class prediction, the outcomes are always divided into four categories: (a) normal samples are correctly predicted as normal (true positives: TP), (b) normal samples are incorrectly predicted as cancer (false negative: FN), (c) cancer samples are correctly predicted as cancer (true negative: TN) and (d) cancer samples are incorrectly predicted as normal (false positive: FP). Then we calculate the following performance measures based on these performance measures:

True positive rate (TPR) = TP / TP + FN, False positive rate (FPR) = FP / (FP + TN), True negative rate (TNR) = TN / (TN + FP) and area under the receiving operating characteristics (ROC) curve, AUC = (TPR + TNR) / 2.

### Patient Classification through Robust SAM Approach

Gene expression datasets are often contaminated by outliers due to several steps involve in the data generating process from hybridization of DNA samples to image analysis [16]. If outliers are present in the dataset then the results of the downstream analysis might be changed. Despite the popularity of the statistical FS methods (t-test or SAM), they are sensitive to outliers. Therefore, in this paper, we used robust SAM [11] as a feature selection method to select the smaller number of informative features to train the classifiers Figure 4. The detail procedure of patient classification is as follows:

1) Apply the robust SAM approach in the GED to select the informative features or DE genes using the p-values. 2) Adjust the p-values for multiple testing corrections using Benjamini-Hochberg method. Then arrange the adjusted p-values in ascending order. 3) Select first T < max (n1, n2) genes out of G genes as top DE genes from the training dataset. Here, n1 and n2 are the number of patient in the normal and cancer group, respectively. G is the total number of gene in the dataset. 4) Estimate the parameters of the classifiers using the expressions of these top T DE genes based on training dataset. 5) Select the expressions of top T DE genes from test dataset to obtain the reduced test dataset. 6) Finally, classify the patients of the test dataset into one of two groups (normal/cancer).

### Dataset

#### Simulated Gene Expression Dataset

We generate the simulated gene expression dataset from the following model as described in Table 1. In this table g1 and g2 represents the up-regulated and down-regulated DE gene group, respectively and g3 represents the EE gene group. We generated gene expression profiles of G=10,000 genes, with k=2 groups (normal/cancer). We considered 100 datasets for both small (N1=N2=10) and large (N1=N2=40) sample cases, respectively. Each dataset for each case represents the gene expression profiles of G=10,000 genes with N= (N1+N2) samples. We set the values of the parameter d as 2 and σ2 = 0.1. Among the expression of 10,000 genes for each datasets we divided these expressions in to two groups (expressions of important features or DE genes, 200 and expressions of the unimportant features or EE genes, 9800). We randomly divided each of the 100 datasets into two independent datasets to construct the training and test dataset such that training and test datasets consist of n1 = N1 / 2 samples in normal and n2 = N2 / 2 samples in cancer group.

#### Real Gene Expression Dataset

This dataset consists gene expression profiles of 6,500 human genes collected from 40 tumor and 22 normal colon tissue samples were analyzed with an Affymetrix technology [17]. Among the 6,500 genes, the highest minimal intensity across the samples with 2000 genes was selected for the further analysis. This dataset can also be downloaded from the R-package ''plsgenomics''.

## Result and Discussion

To demonstrate the performance of the robust SAM in a comparison of classical t-test and SAM for both simulated and real gene expression datasets, we pick up four popular classifiers: linear discriminant analysis (LDA) [12], K-nearest neighborhood (KNN) [13], support vector machine (SVM) [14] and naive Bayes classifier [15]. We used three R packages for the four classifiers: MASS for LDA, kknn for KNN, e1071 for SAM and naive Bayes. The performance measure AUC was computed for each of the classifiers using ROC R package. All R packages are available in the comprehensive R archive network (cran) or bioconductor.

### Performance Evaluation Based on Simulated Gene Expression Dataset

**Figure 1:**
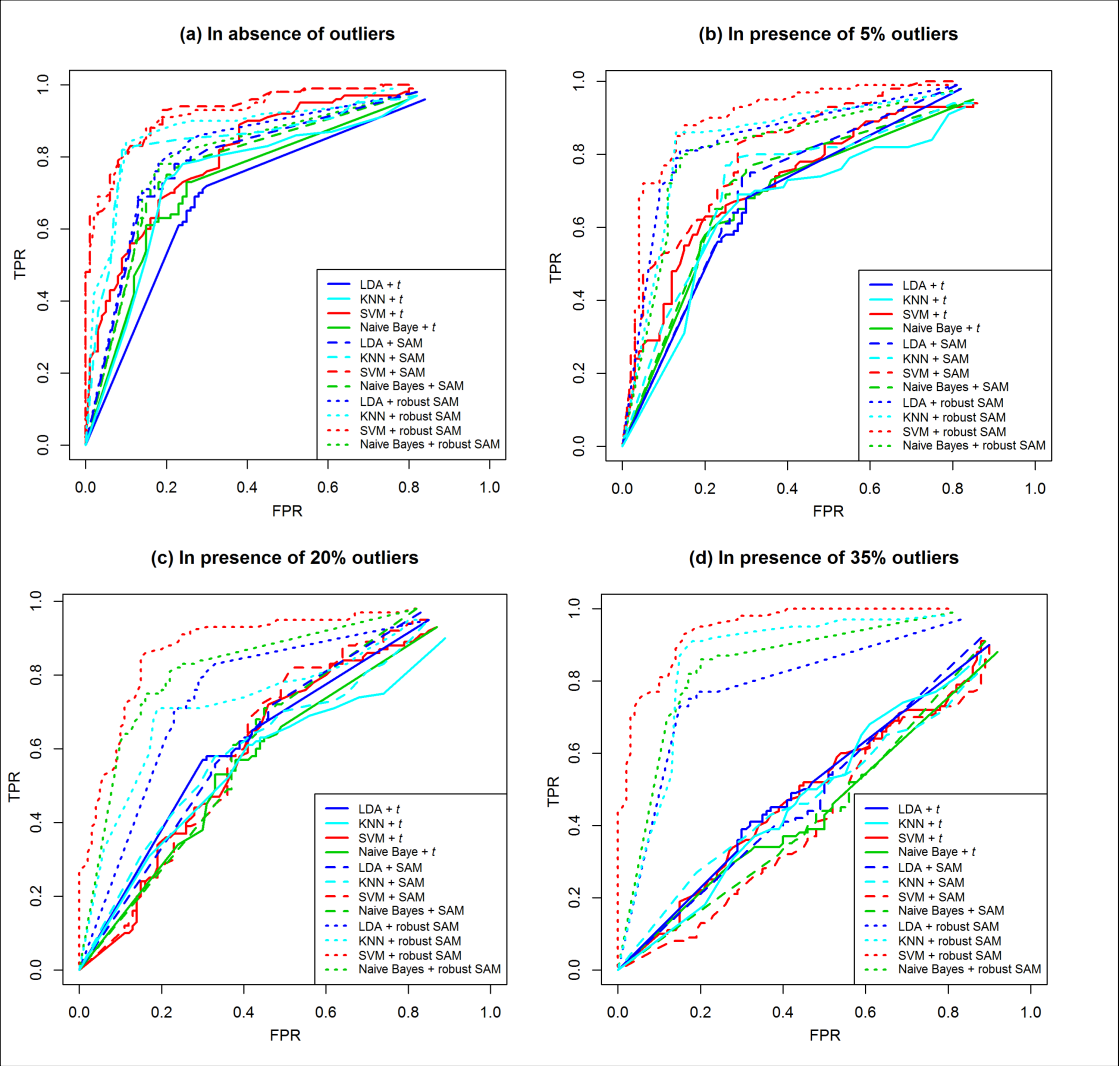
Performance evaluation using tests ROC curve produced by four classifiers for simulated dataset with sample size (n1=n2=5). (a) In absence of outliers. (b) In presence of 5% outliers. (c) In presence of 20% outliers. (d) In presence of 35% outliers.

To investigate the performance of the robust SAM in a comparison of classical t-test and SAM, we employed these three FS methods to identify the 200 informative features (DE genes) from each of the 100 simulated training datasets as described above. We select the top T < max (n1, n2) DE genes obtained from the three methods by ranking the adjusted p-values in ascending order. The adjusted p-values were obtained using Benjamini- Hochberg method. The expressions of these top T (10 or 40) detected DE genes are then used to train the four popular classifiers (LDA, KNN, SVM and naive Bayes) to predict the patients/samples class. We computed four performance measures such as TPR, TNR, FPR and AUC based on reduced training and test datasets using the four classifiers. A method is said to be good performer if it produces larger values of TPR, TNR, AUC and smaller values of FPR. To show the effect of outliers in the three FS methods, we randomly corrupted 5%, 20% and 35% genes by a single outlier in the training datasets. Here, values of outliers are considered as larger than the maximum value of the expressions. The Figure 1 represents the test ROC curve produced by the four classifiers using the average values of 100 estimated FPR and TPR based on 100 training and test datasets for small-sample case (n1=n2=5). From this figure we observe that, in absence of outliers, all the four classifiers (LDA, KNN, SVM and naive Bayes) performed well when feature selection is carried out from SAM and robust SAM. In this case classical t-test performed slightly worse than the SAM and robust SAM. But in presence of outliers (5%, 20% and 35%), the performances of all the four classifiers deteriorate by producing lower values of AUC (< 0.80) with classical t test and SAM. Four classifiers performed well with robust SAM (AUC > 0.85) for same conditions. On the other hand, for large-sample (n1=n2=20) case in absence and presence of 5% and 20% outliers, all the four classifiers produces almost similar values of AUC with classical ttest, SAM and robust SAM. But in presence of 35% outliers, in this case, these four classifiers performed well only when FS is carried out from robust SAM (see Table 4 in supplementary file).

### Performance Evaluation Based on Real Colon cancer Gene Expression Dataset

**Figure 2:**
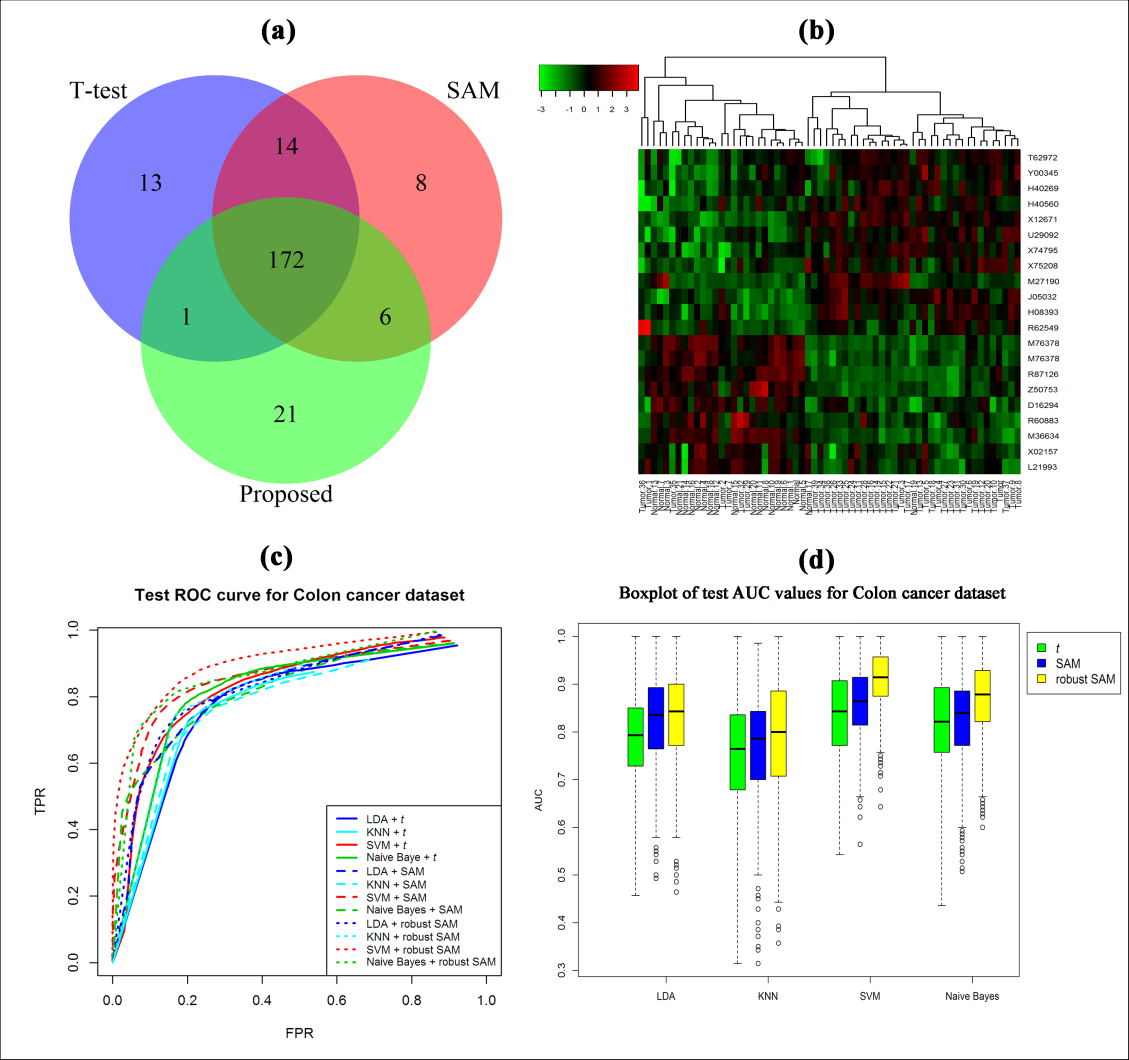
Comparison of the DE genes detected by t-test, SAM and robust SAM for the Colon cancer dataset. (a) Venn diagram of DE genes detected by t-test, SAM and robust SAM. (b) Heatmap of 21 DE genes identified by the robust SAM. (c) Test ROC curve produced by four classifiers using the expression values of 13, 8 and 21 DE genes identified by t-test, SAM and robust SAM, respectively. (d) Boxplot of AUC values estimated by the four classifiers using t-test, SAM and robust SAM. 1000 trials were performed to obtain this result.

**Figure 3:**
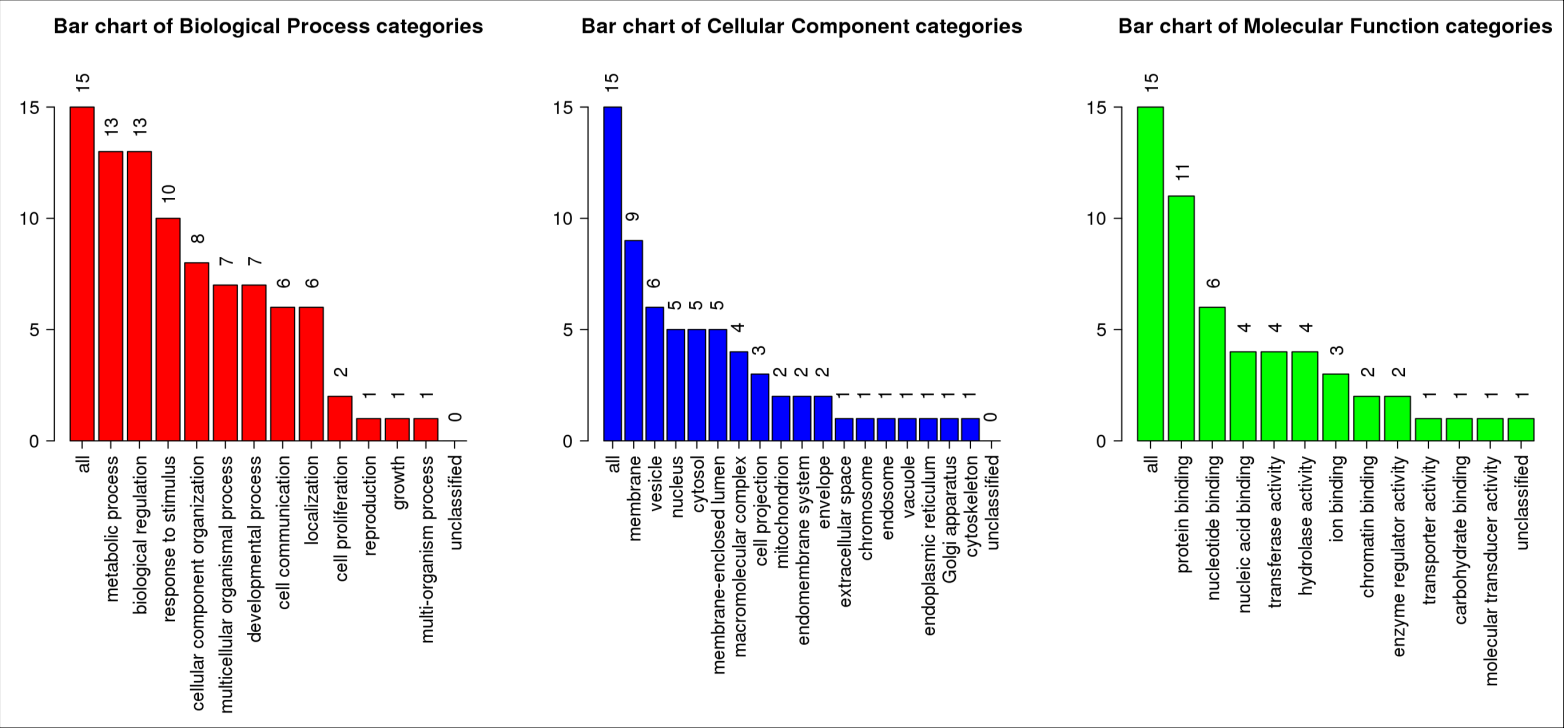
Functional annotation of 21 DE genes identified by the robust SAM. Frequency distribution of biological process, cellular component and molecular function categories for 15 DE genes identified by robust SAM. KEGG identified 15 genes out of 21 DE genes using in WebGestalt software.

**Figure 4:**
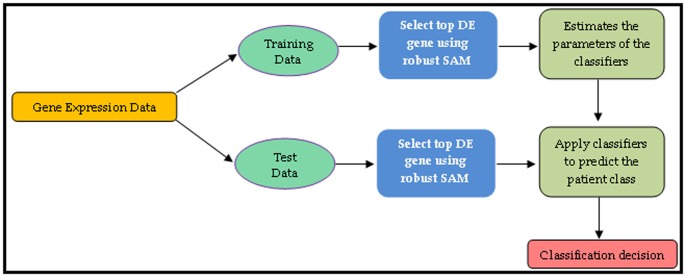
Computational pipeline for patient classification through robust SAM

To demonstrate the performance of robust SAM in a comparison of classical t-test and SAM, we employed these methods in the real Colon cancer dataset to detect the DE genes. We select top 200 DE genes by ranking the adjusted p-values. The adjusted pvalues were obtained using Benjamini-Hochberg method. The detecting performance of top 200 DE genes using classical t-test, SAM and robust SAM is shown in a Venn diagram of Figure 2a. From this figure we notice that there are additional 13, 8 and 21 DE genes identified by classical t-test, SAM and robust SAM, respectively. The Figure 2b shows heatmap using the 21 genes detected by the robust SAM. We can clearly observe from this heatmap that these 21 genes have classified the samples into two groups (normal and cancer). To investigate the classification performance of the four classifiers (LDA, KNN, SVM and naive Bayes) using the expressions of additional 13, 8 and 21 genes detected by the three methods, we randomly divided this Colon cancer dataset into two datasets (training dataset and test dataset) such that each dataset contains same number of samples. Then we computed the four performance measures (TPR, TNR, FPR and AUC) using the four classifiers. The average values of AUC are summarized in Table 2. From this table we clearly notice that the performance of all the classifiers is improved using robust SAM. We also observe that SVM and naive Bayes classifiers performed better than the LDA and KNN. The test ROC curve shown in Figure 2c also supports the results of Table 2. The Figure 2d shows the boxplot of test AUC values estimated by the four classifiers for Colon cancer dataset using classical t-test, SAM and robust SAM. This plot also supports the results of Table 2. To elucidate the molecular functions and KEGG pathways of these additional 21 genes, we used WebGestalt software package [18]. The Figure 3 shows the bar chart of the biological process, cellular component and molecular function categories. In this figure there are 15 genes out of 21 genes involved in the three categories. The top ten KEGG pathways for additional 21 genes detected by the robust SAM is summarized in Table 3. We found that DNA replication pathway is the highest enriched pathway.

## Conclusion

Patient classification into various sources of population of training dataset is very popular in GED. t-test and SAM are the popular FS methods for patient classification using GED. However, both of them suffer from outliers. To prevail over the problems of classical t-test and SAM, robust SAM using the minimum β-divergence estimators was proposed [11]. In this paper, we employed robust SAM as a FS method along with classifiers. From a real Colon cancer dataset we identified additional 21 DE genes by robust SAM and we found that these genes are involved in some important pathways related to cancer disease. Then we apply the expressions of these 21 genes in the classification and reveal that the classification performances improve using these genes. Moreover, we notice that SVM and naive Bayes classifiers performed better compare to the LDA and KNN.

## Figures and Tables

**Table 1:** Simulated gene expression data generating model for k=2 groups

Gene Group	Patients	
Normal (N1)	Cancer(N2)
g1	-d + N(0, σ 2)	d + N(0, σ 2)
g2	d + N(0, σ 2)	-d + N(0, σ 2)
g3	d + N(0, σ 2)	d + N(0, σ 2)

**Table 2:** Performance evaluation using test AUC values for Colon cancer dataset

Classifiers	Feature Selection Methods		
t-test	SAM	Robust SAM
LDA	0.788	0.828	0.834
KNN	0.745	0.766	0.787
SVM	0.839	0.862	0.914
Naive Bayes	0.817	0.825	0.873

The performance measure AUC values were estimated using four classifiers (LDA, KNN, SVM and naive Bayes), based on 13, 8 and 21 DE genes identified by classical t-test, SAM and robust SAM approach, respectively.			

**Table 3:** KEGG pathways for 21 DE genes detected using robust SAM for Colon cancer dataset

KEGG ID	Name of Pathways	No of Gene	Adjusted p-values
hsa03030	DNA replication	2	2.29E-01
hsa00230	Purine metabolism	3	2.29E-01
hsa00511	Other glycan degradation	1	9.70E-01
hsa03430	Mismatch repair	1	9.70E-01
hsa00062	Fatty acid elongation	1	9.70E-01
hsa03410	Base excision repair	1	9.70E-01
hsa05166	HTLV-I infection	2	9.70E-01
hsa03440	Homologous recombination	1	9.70E-01
hsa00071	Fatty acid degradation	1	9.70E-01
hsa03420	Nucleotide excision repair	1	9.70E-01

KEGG terms that are significantly enriched in the 15 Colon cancer related genes detected by the robust SAM. The p-values were calculated using hypergeometric test and then adjusted by Benjamini-Hochberg method for multiple testing corrections. 15 genes out of 21 genes were mapped using the KEGG map in WebGestalt sortware.			

**Table 4:** Performance evaluation using test AUC values estimated by the four classifiers for simulated dataset

Feature Selection (FS)	For large-sample case (n1= n2=20)							
In absence of outliers				In presence of 5% outliers			
LDA	KNN	SVM	naive Bayes	LDA	KNN	SVM	naive Bayes
t-test	0.983	0.96	0.992	0.985	0.964	0.952	0.982	0.961
SAM	0.985	0.972	0.993	0.992	0.95	0.962	0.984	0.973
robust SAM	0.98	0.963	0.991	0.993	0.982	0.963	0.99	0.992
FS	In presence of 20% outliers				In presence of 35% outliers			
	LDA	KNN	SVM	naive Bayes	LDA	KNN	SVM	naive Bayes
t-test	0.935	0.928	0.952	0.947	0.6	0.66	0.53	0.62
SAM	0.93	0.915	0.949	0.933	0.621	0.632	0.562	0.633
robust SAM	0.98	0.962	0.99	0.991	0.974	0.952	0.982	0.987

In this table performance measure test AUC values were estimated by the four classifiers (LDA, KNN, SVM and naive Bayes) based on top 200 DE genes for large (n1= n2=20) sample cases.
